# Effect of Ca-P compound formed by hydrothermal treatment on biodegradation and biocompatibility of Mg-3Al-1Zn-1.5Ca alloy; *in vitro* and *in vivo* evaluation

**DOI:** 10.1038/s41598-017-00656-0

**Published:** 2017-04-06

**Authors:** Yu-Kyoung Kim, Yong-Seok Jang, Young-Hee Lee, Ho-Keun Yi, Tae-Sung Bae, Min-Ho Lee

**Affiliations:** 1 Department of Dental Biomaterials and Institute of Biodegradable Materials, Institute of Oral Bioscience and School of Dentistry (plus BK21 program), Chonbuk National University, Jeon Ju, South Korea; 2 Elcu bio Co. Ltd, Yuseong-gu, Daejeon South Korea; 3 Department of Oral Biochemistry, Institute of Oral Bioscience, School of Dentistry and plus BK21 program, Chonbuk National University, Jeon Ju, South Korea

## Abstract

Chemical combinations of Ca-P produced via plasma electrolytic oxidation (PEO) and a hydrothermal treatment were fabricated to improve the initial corrosion resistance and biocompatibility of a biodegradable Mg-3Al-1Zn-1.5Ca alloy. For the formation of an amorphous calcium phosphate composite layer on the surface of a magnesium alloy, a PEO layer composed of MgO and Mg_3_(PO_4_)_2_ was formed by PEO in electrolytes containing preliminary phosphate ions. During the second stage, a thick and dense Ca layer was formed by Ca electrodeposition after PEO. Finally, a hydrothermal treatment was carried out for chemical incorporation of P ions in the PEO layer and Ca ions in the electrodeposition layer. The amorphous calcium phosphate composite layer formed by the hydrothermal treatment enhanced osteoblast activity and reduced H_2_O_2_ production, which is a known stress indicator for cells. As a result of co-culturing osteoblast cells and RAW 264.7 cells, the formation of amorphous calcium phosphate increased osteoblast cell differentiation and decreased osteoclast cell differentiation. Implanting the alloy, which had an amorphous calcium phosphate composite layer that had been added through hydrothermal treatment, in the tibia of rats led to a reduction in initial biodegradation and promoted new bone formation.

## Introduction

Magnesium is a dietary element with the highest concentration in the body after potassium^1^. It is present at an amount of 20–28 g in the body, of which 60% exists in the bones, 27% in the muscles, and 1% in the extracellular fluid. The remainder resides in soft tissues and other body fluids. Furthermore, Mg has a low density and mechanical properties similar to the properties of natural bone because it is absorbed readily by the body. Therefore, it is used widely in various types of implants^2^. However, due to its low corrosion resistance, it has limited applications. Extensive efforts are being made to improve its compatibility by increasing its corrosion resistance through various surface treatments and by loading it with drugs or materials similar to bone^3, 4^.

Plasma electrolytic oxidation (PEO) using an alkali base as the electrolyte, such as NaOH or KOH, is a surface-treatment method for controlling the electrical conditions and increasing the corrosion resistance of biodegradable Mg^5–7^. It has been reported that the addition of a phosphate layer improves the corrosion resistance of Mg. PEO with Na_2_HPO_4_ on Mg results in better corrosion resistance than anodizing with Na_2_SiO_3_^8^. In particular, the phosphate coating reacts with the Ca ions in the body fluids, which combines with the PO_4_ ions in the oxide film and results in the deposition of amorphous calcium phosphate on the surface^9^. To induce the chemical combination of P and Ca artificially, PEO treatments are carried out using synthetic electrolytes, such as Ca(NO_3_)_2_ and NH_4_H_2_PO_4_^10^, Ca(OH) and Na_3_PO_4_·12H_2_O^11^, or nicotinic acid and calcium phosphate^12^. However, if the Ca and P containing synthetic electrolytes are added to an alkali basic electrolyte for plasma anodization, Ca(OH)_2_ is deposited on the electrolytes. It is difficult to manufacture electrolytes quantitatively.

Ca electrodeposition on the phosphate layer is a chemical combination process that results in the formation of deposits similar to osseous tissue. By controlling the deposition time and voltage, it is possible to adjust the type of film formed and its thickness^13^. Non-surface-treated Mg alloys usually are difficult to induce Ca–P nucleation in a physiological environment or supersaturated aqueous solutions. The proper surface treatment and adequate post-processing are essential for bioactivity^14^. Thus, the electrodeposition of Ca on the microporous layer formed by a PEO treatment should increase the biocompatibility of Mg in body fluids.

The amorphous Ca and P deposited on films in this manner exist in the form of various apatites, including hydroxyapatite (HA; Ca_10_(PO_4_)_6_(OH)_2_). It was reported that these materials have chemical compositions similar to bone tissue and can also combine well with bone^15^. Given these facts, an additional hydrothermal treatment after the PEO treatment can be used to deposit a large amount of HA. This treatment would improve the probability of the implant combining with bone. The films formed by the hydrothermal treatment at low temperatures (typically 100–200 °C) exhibit many desirable qualities, such as high purity, high thickness, and good adhesion^16^.

In other studies, hydrothermal treatment has been used to form functional films, such as BaTiO_3_, SrTiO_3_, and lead zirconate titanate on the surfaces of ceramics and the materials used in sensors^17, 18^. There have also been a number of studies on inducing the partial crystallization of Ca and P that exist in the oxide layer of Ti implants through a hydrothermal treatment after anodization using an electrolyte containing phosphates and Ca ions for improving the bone combination characteristics of the implants^19, 20^. In this study, the electrodeposition of Ca was performed on a PEO coating on a Mg-3Al-1Zn-1.5Ca alloy to form an oxide and Ca double layer. And we investigated whether the chemical combination of P and Ca through the hydrothermal treatment improved the corrosion resistance and biocompatibility of the alloy.

## Results

**Figure 1:**
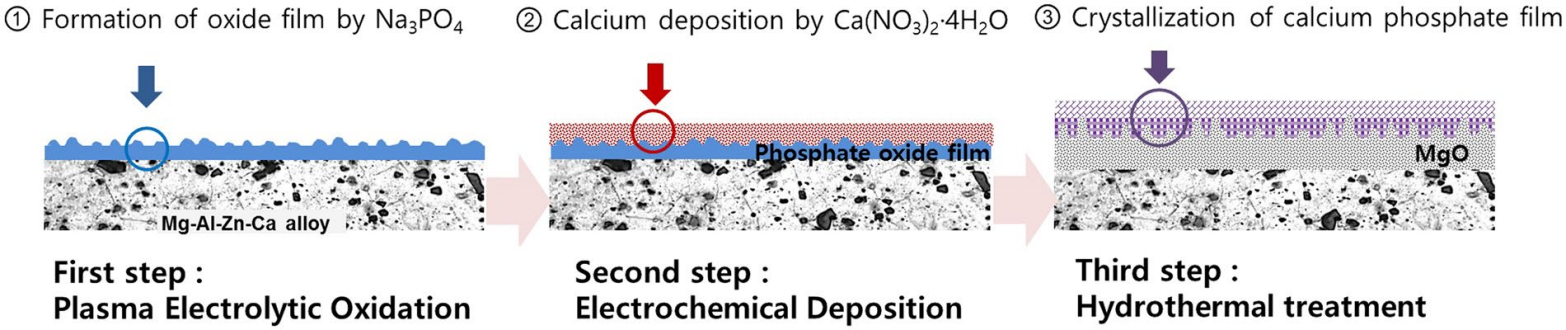
Schematic of surface-treatment process.

In this study, a double-layer was formed on the surface of Mg-3Al-1Zn-1.5Ca alloy by PEO treatment and the deposition of Ca. Through the hydrothermal treatment, the chemical combination of the electrodeposited Ca and P could be induced successfully. Figure 1 shows a schematic of the steps involved in the surface-treatment process.

Before surface treatment, the physical characteristics of the alloy were examined to investigate the effects of the hydrothermal treatment. When the hydrothermal treatments were conducted at 120 °C and 150 °C, the grain size of the material was similar to that in the untreated group. However, if the material was hydrothermally treated at more than 180 °C, a distinct magnesium oxide layer was detached. Therefore, the group subjected to the hydrothermal treatment at 180 °C was excluded from the study.

**Figure 2:**
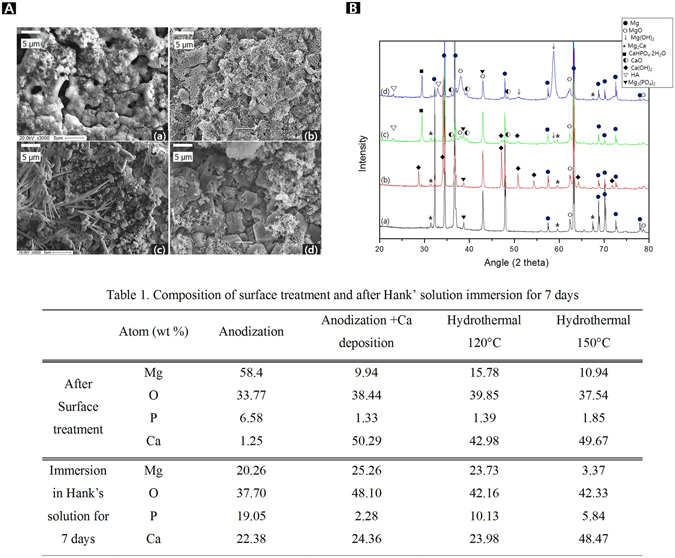
Surface morphology (**A**) and XRD patterns (**B**) of (a) anodization on AZ31B-1.5Ca (b) Ca deposition after anodizing (c) hydrothermal treatment at 120 °C, (d) hydrothermal treatment at 150 °C, and Compositions of specimens after the surface treatment and after immersion in Hank’ solution for 7 days, and composition of surface after treatment and SBF immersion (**C**).

Figure 2A shows SEM images of the surfaces after the surface treatment. The results of the EDS analysis of the surface layers (that is, the components of the surface layers) are listed in Fig. 2C. The PEO treatment (Fig. 2A(a)) involved the generation of a spark on the sample surface, which resulted in the formation of an oxide film with irregular pores. The multi-layered film of crystal blocks with a sponge-like structure was formed in the porous oxidation film by the Ca deposition (Fig. 2A(b)). The hydrothermal treatment at 120 °C resulted in two types of coexisting crystals, acicular and spherical crystals. The hydrothermal treatment at 150 °C resulted in the formation of polyhedral crystals, which consisted of clusters of smaller particles that were acicular and had petal-like structures. Figure 2C shows that the PEO treatment involving the addition of Na_3_PO_4_ resulted in a high oxygen concentration, and P was detected in these samples because of the Na_3_PO_4_ in the electrolyte. Finally, it was confirmed that Mg and Ca were the constituent elements of the deposited material. After the electrodeposition process, the Ca composition increased to 50.29 wt%. However, there was no change in the oxygen concentration, while the amounts of Mg and P decreased slightly.

The phase analysis was performed using XRD measurements (Fig. 2B), which confirmed that MgO and Mg_3_(PO_4_)_2_ were formed by the anode oxidation process (Fig. 2B(a)). After electrodeposition, new peaks related to CaO and Ca(OH)_2_ were observed. Finally, after the hydrothermal treatments, the Ca(OH)_2_-related peak was not observed. Instead, peaks related to a calcium apatite, such as CaHPO_4_·2H_2_O and hydroxyapatite (Ca_10_(PO_4_)_6_(OH)_2_), were formed. In particular, the patterns of the samples subjected to the hydrothermal treatment at 150 °C (d) showed peaks that could be attributed to HA and Mg(OH)_2_.

**Figure 3:**
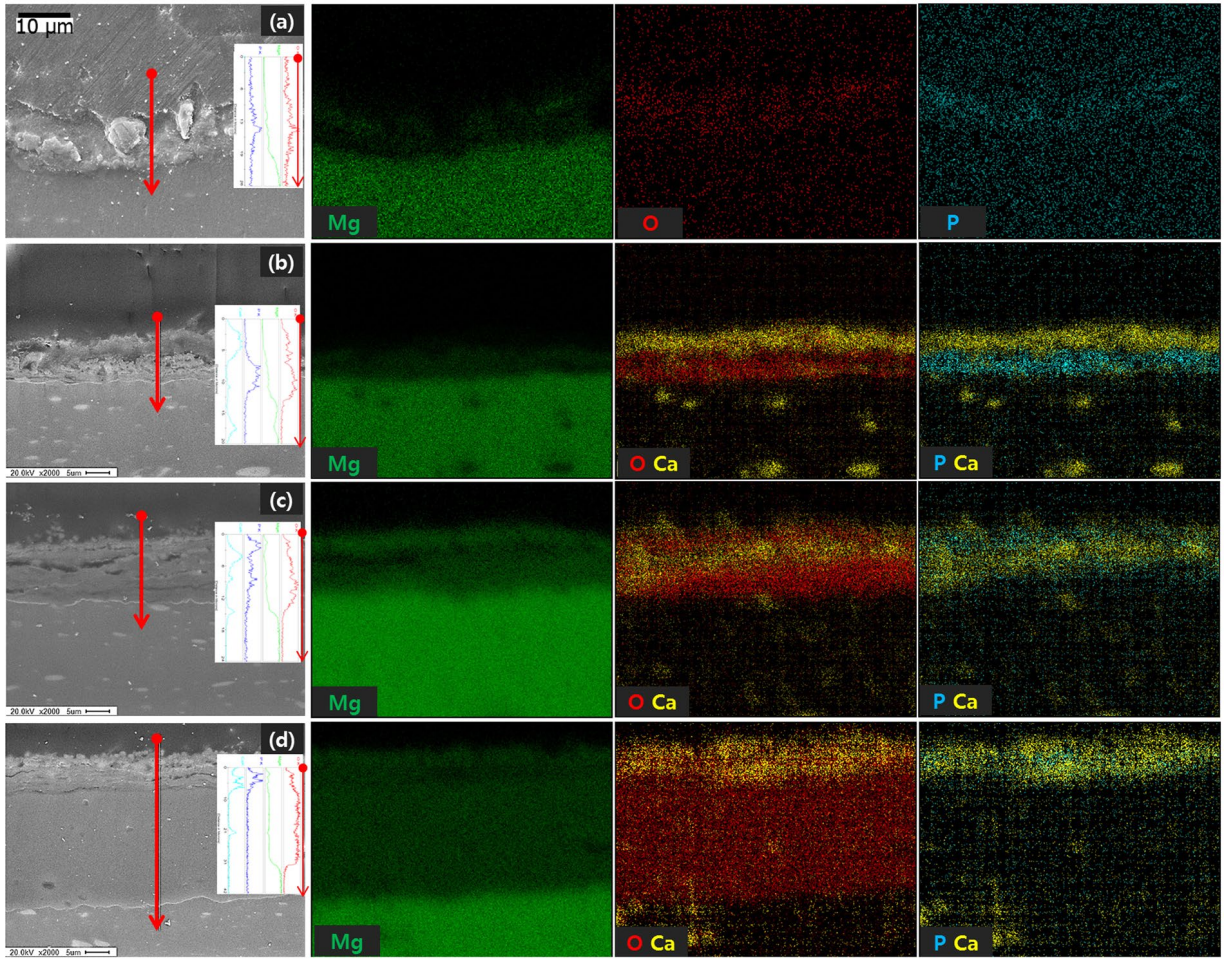
Cross-sectional image and line scans of surfaces film after (**a**) anodization, (**b**) Ca deposition post anodization anodizing, (**c**) hydrothermal treatment at 120 °C, and (**d**) hydrothermal treatment at 150 °C.

Figure 3 shows cross-sectional images and elemental maps of the films formed on the samples. The thickness of the film formed by oxidization with Na_3_PO_4_ was 9.21 ± 0.35 μm (Fig. 3(b)). The surface of the Ca deposition group (b) was covered with 9.1 ± 0.408 μm thickness. The thickness of this film was a little different from the thickness formed on the previous sample. However, the film was clearly double layered because Ca was deposited on an oxide layer composed of P. This oxide layer (Fig. 3(b)) had a thickness that was half the thickness of the layer formed by anode oxidation (a), but it had a higher ion density. The hydrothermal treatment at 120 °C (c) resulted in the formation of film with a greater thickness, 12.25 ± 0.78 μm. Furthermore, both P and Ca were mixed and present at a high concentration along with O in the film. The hydrothermal treatment at 150 °C (d) formed an oxide layer with a significantly increased thickness (29.25 ± 0.18 μm). However, the thickness of the Ca-P layer was reduced, and a thick MgO layer was formed between the Ca-P layer and the Mg matrix.

**Figure 4:**
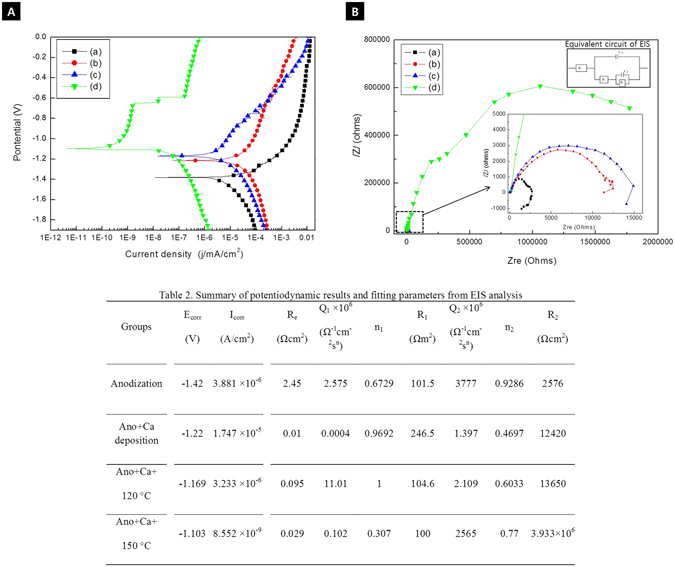
Potentiodynamic polarization curves (**A**) and Nyquist plots from EIS (**B**) as determined of Mg-3Al-1Zn-1.5Ca alloy (a) after anodization, (b) Ca deposition post anodizing, (c) hydrothermal treatment at 120 °C, and (d) hydrothermal treatment at 150 °C, and summary of potentiodynamic and fitting parameter from EIS test (**C**).

Figure 4 shows the results of corrosion resistance evaluations, which were performed based on potentiodynamic polarization (A) and EIS (B). The corrosion potential in the case of the sample in Fig. 4A(a) was the lowest (−1.42 V), and the corrosion current density was 3.881 × 10^−5^ A/cm^2^, which indicated that this sample exhibited poor corrosion resistance. The corrosion potential for the samples subjected to Ca deposition (b) and the hydrothermal treatment at 120 °C (c) were −1.22 and −1.169 V, respectively, and their corrosion current densities were 1.747 × 10^−5^ and 3.233 × 10^−6^ A/cm^2^. Therefore, the corrosion resistance increased after the hydrothermal treatment. In particular, the corrosion current density of the samples treated hydrothermally at 150 °C (d) was 8.552 × 10^−9^ A/cm^2^, which was used to fabricate the samples with the highest corrosion resistance. In the Nyquist plot (Fig. 4B), the semicircles were fitted using equivalent circuit models (ZSimpWin software). The electrolyte resistance values (*R*_e_) were not significantly different in the samples hydrothermally treated at 150 °C (d). The polarization resistance (*R*_p_) was determined by adding the coating resistance (*R*_1_) and the charge-transfer resistance (*R*_2_). In the group that was hydrothermally treated at 150 °C, the charge-transfer resistance (R_2_) was greatly increased because thick and dense MgO/Mg(OH)_2_ formed inside the Ca-P layer. The EIS results showed the same tendency as the potentiodynamic polarization measurements. Anodization with Na_3_PO_4_ (a) and Ca deposition (b) resulted in films with similar thicknesses, but the corrosion resistance increased sharply after Ca deposition.

**Figure 5:**
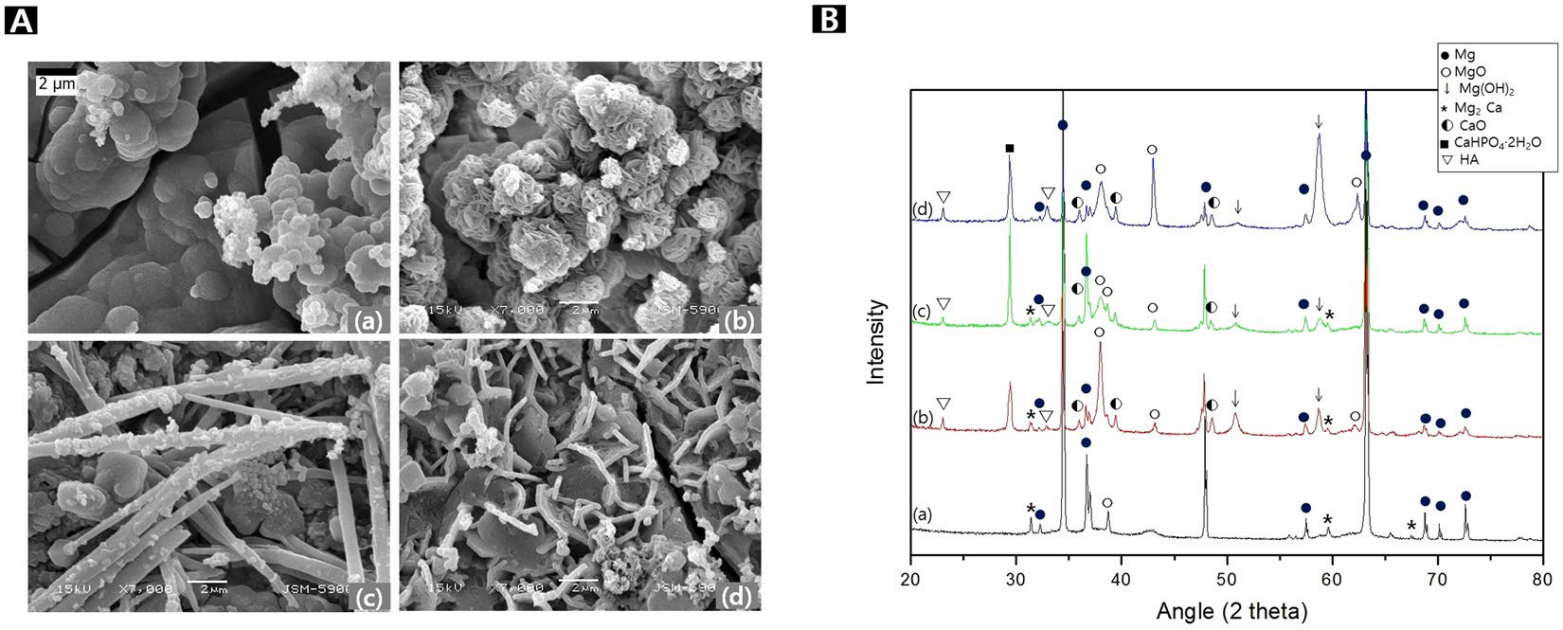
Surface morphologies (**A**) and XRD patterns (**B**) after immersion in Hank’s solution for 7 days (a) after anodization, (b) Ca deposition post anodization, (c) hydrothermal treatment at 120 °C, and (d) hydrothermal treatment at 150 °C.

A shown in Fig. 5A, the sample subjected to anodization by Na_3_PO_4_ (a) formed cracks as a phase with spherical particles was deposited on it. For Ca deposition (b), the sponge-type crystals reacted with the ions in Hank’s solution for 7 days, which turned into budding-flower-like crystals. On the surfaces of the acicular structures formed by the hydrothermal treatment at 120 °C (c), nanosized spherical crystals grew, but no cracks were formed. In the case of the sample subjected to the hydrothermal treatment at 150 °C (d), the petal-like structures were transformed into smaller acicular ones. The components of the surface layers of the various samples are listed in Fig. 2C. Anodization by Na_3_PO_4_ (a) did not result in a change in composition. On the other hand, the amounts of Ca and P increased and Mg decreased slightly, and the amount of calcium apatite increased. Ca deposition (b) resulted in a decrease in the amount of Ca and an increase in the amounts of O and Mg. These changes mean that the Ca ion was eluted by combining oxide film dissolution and the exposed substrate and oxygen due to the progress of corrosion. The hydrothermal treatment at 120 °C (c) resulted in an increase in the amount of P and a decrease in the amount of Ca. However, the concentrations of Mg and O remained unchanged. This trend indicated that this sample reacted with the Hank’s solution, which absorbed P and discharged Ca and resulted in the formation of calcium apatite. On the other hand, the hydrothermal treatment at 150 °C (d) also resulted in a similar Mg concentration. Furthermore, Ca and P did not change significantly, and there was no difference in the amount of calcium apatite formed because of deposition from the Hank’s solution, and the stable oxide film remained intact. Figure 5B showed the XRD patterns of the crystals formed on the surfaces of samples after immersion in Hank’s solution for 7 days. For all the groups, the intensity of the Mg-related peak was reduced and the MgO-related peak increased. The Ca(OH)_2_ peak disappeared for the Ca deposition group, while peaks related to CaHPO_4_·2H_2_O and HA were observed. The CaHPO_4_·2H_2_O peak in the group that was hydrothermally treated at 120 °C increased sharply in intensity.

**Figure 6:**
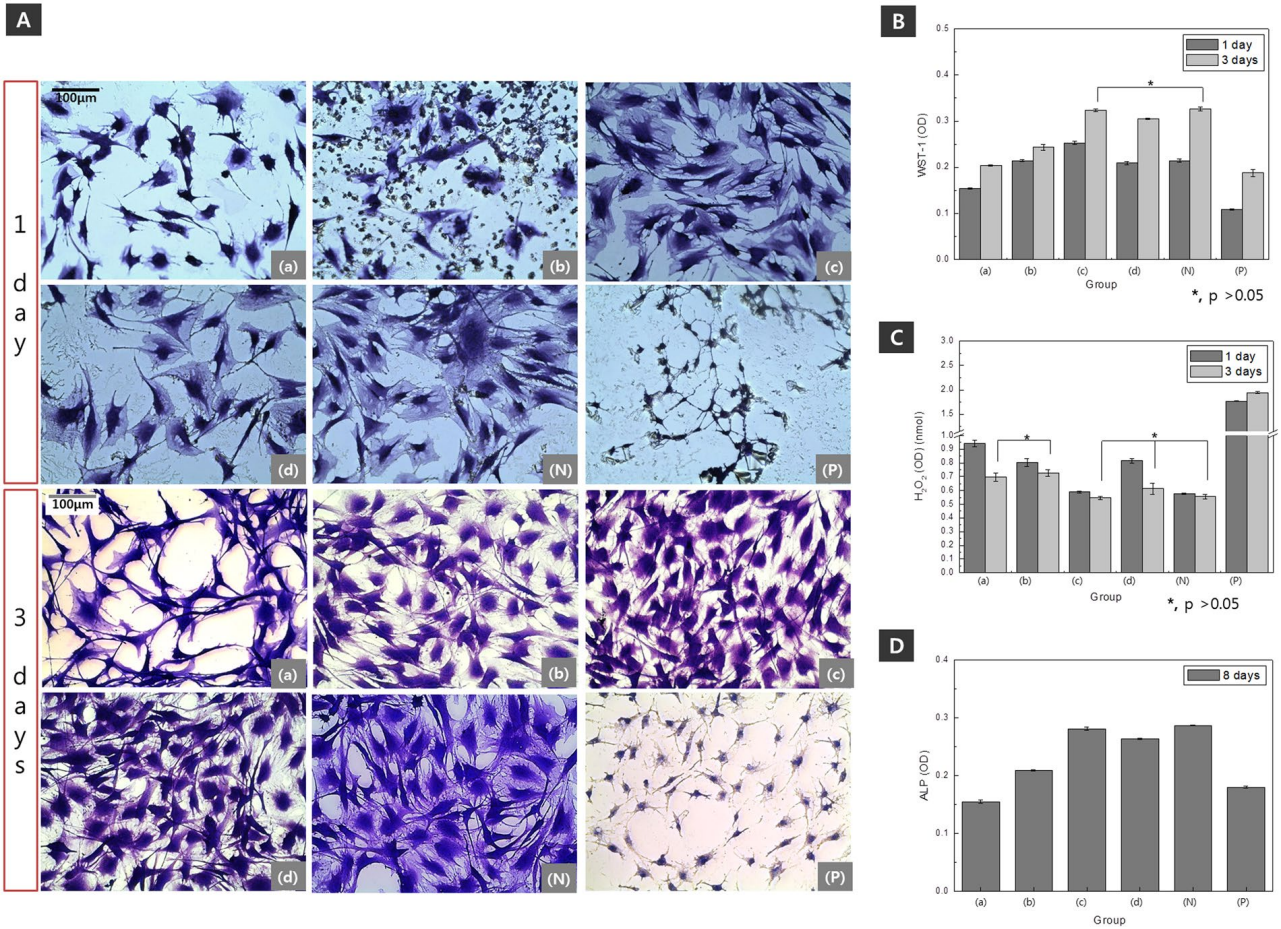
Cell morphology (**A**), WST-1 cell proliferation assay results (**B**), H_2_O_2_ concentration (**C**), and ALP activity (**D**) of MC3T3-E1 cells after 1, 3, and 8 days of culturing of Mg-3Al-1Zn-1.5Ca alloy (a) after anodization (b) Ca deposition post anodization, (c) hydrothermal treatment at 120 °C, and (d) hydrothermal treatment at 150 °C.

To examine the effects of osteoblast proliferation, the samples were eluted in the appropriate medium, and the cells were cultivated for 1 and 3 days. The nuclei and cytoplasm of the surviving cells were observed (Fig. 6A). In particular, Fig. 6A(a) shows that only a few areas contained the cytoplasm, and there was a significant decrease in the cell unit size and low cell growth after 1 and 3 days. The group that was hydrothermally treated at 150 °C and the normal group (N) showed similar cell growth rates, while the group that was hydrothermally treated at 120 °C showed the highest cell viability. After 3 days, the two hydrothermally treated groups (c and d) showed cell growth rates that were higher than the normal group (N). However, the cells were still shrunken compared to cells in the normal group. The results of quantitative evaluations of the cell survival rate using the WST-1 assay (Fig. 6B) showed that after 1 day, the group that was hydrothermally treated at 120 °C showed the highest cell growth, while the anodized group (a) had the lowest cell viability. After 3 days of culturing, the hydrothermally treated groups showed excellent cell growth. In particular, the group that was hydrothermally treated at 120 °C exhibited the highest cell growth rate. The results of the measurements of the amount of H_2_O_2_ produced in the cultured medium (Fig. 6C), which were performed to evaluate the initial oxidation stress, showed that anodization by Na_3_PO_4_ (a) resulted in the highest amount of H_2_O_2_ after 1 day of culture. All the groups exhibited lower production rates for H_2_O_2_ after 3 days. In addition, the groups in (a) and (b) did not show any significant differences, and all the hydrothermally treated groups had low H_2_O_2_ production rates. ALP is known as an initial indication factor of osteoblast differentiation. The ALP activities of the samples after 8 days were determined through a quantitative analysis (Fig. 6D). The sample anodized by Na_3_PO_4_ (a) showed the lowest activity, while the sample hydrothermally treated at 120 °C (c) showed the highest activity. In addition, after the hydrothermal treatments, the ALP expression increased markedly, with the results showing the same tendency as the cell proliferation after 3 days.

**Figure 7:**
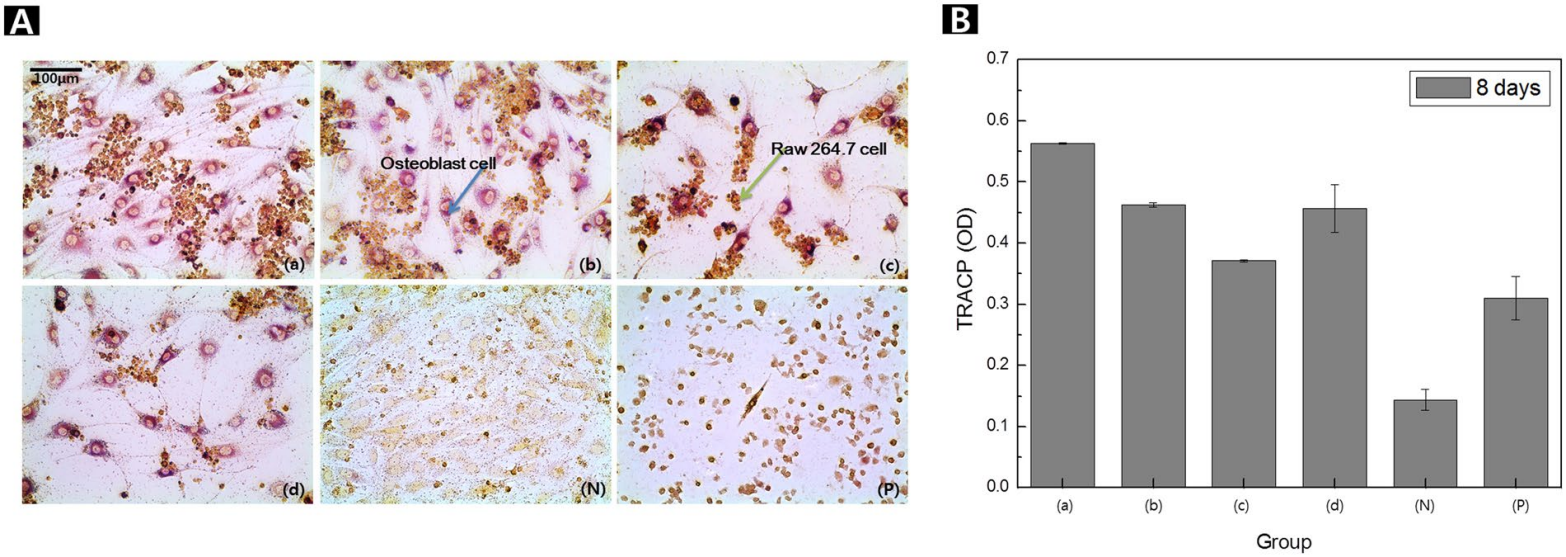
TRAP staining assay results (**A**) and TRACP activity (**B**) of osteoclast cells after co-cultured cell growth for 8 days (a) after anodization, (b) Ca deposition post anodization, (c) hydrothermal treatment at 120 °C, and (d) hydrothermal treatment at 150 °C.

Figure 7A shows the osteoclast cells analyzed using TRAP and TRACP assays after 8 days of co-culture with MC3T3-E1 osteoblast cells and RAW 264.7 cells. The osteoclast expression was evaluated quantitatively. The macrophage cells and osteoblast cells were affected in the neural transmitter from extracted ions of each sample. The anodization group (a) resulted in the highest osteoclast activity and the highest macrophage cell survival rate that positive TRAP cells were dyed red. For the group that was hydrothermally treated at 120 °C (c), the osteoclast activity was the lowest. Furthermore, the normal group (N) showed a low osteoclast survival rate, due to the active differentiation and multiplication of the osteoblast cells, as an absorbance value of the TRACP assay (Fig. 7B).

**Figure 8:**
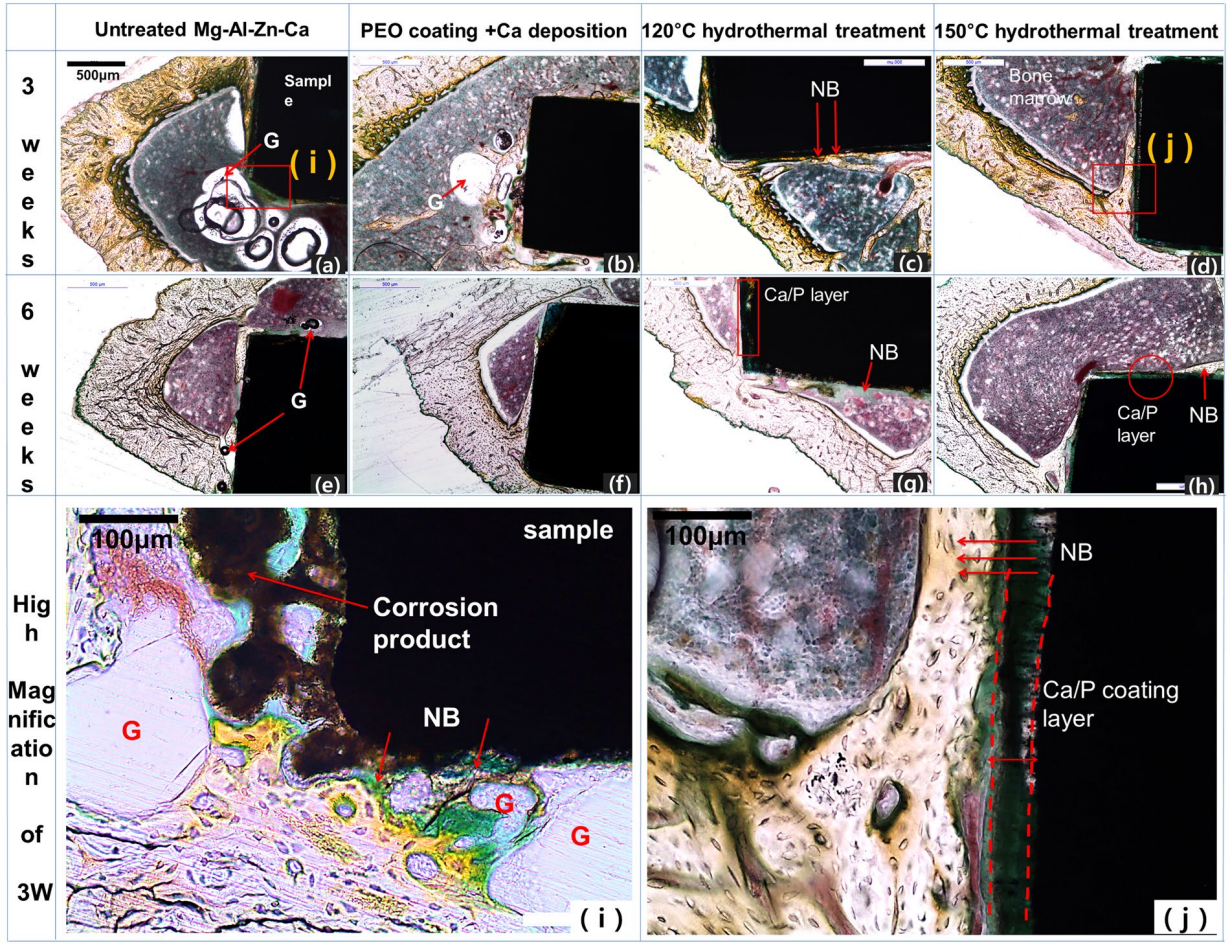
Histological analyses of rat tibia for each group 3 and 6 week after implantation, and high-magnification images of (i) Mg-3Al-1Zn-1.5Ca alloy and (j) after the hydrothermal treatment at 150 °C and implantation for 6 weeks.

Figure 8 shows the results of the histological analyses of the areas of the rat tibia in contact with bone as well as the results for the samples 3 and 6 weeks after implantation in a direction perpendicular to the tibia. For the untreated group (a), a large number of gas bubbles were observed around the bone marrow and on the periphery of the test specimen. This made it difficult for the sample to remain in contact with the bone after 3 weeks. After 6 weeks of implantation, most of the gas bubbles were absorbed with a few remaining in the bone marrow (e). The sample in the anodization with Ca group (b) also contained gas bubbles, but there were fewer bubbles for the untreated group (a). The image in (f) shows a gas bubble (G) that was absorbed in a specimen of the Ca deposition group. It can also be observed that new bone (NB) was formed around the specimen. In the case of the hydrothermally treated groups, no gas bubbles were observed after 3 weeks (c and d). In particular, for the group that was hydrothermally treated at 150 °C, the Ca-P layer remained intact on the magnesium alloy substrate after 6 weeks (h). Figure 8(i and j) shows magnified images of the boundaries of the specimens and bone tissue. The untreated group sample eluted Mg ions in the direction of the bone marrow due to its corrosion rate being higher. As a result, several corrosive particles and gas bubbles were formed in the bone marrow. On the other hand, a distinct Ca-P layer was observed in the samples that were hydrothermally treated at 150 °C. And dense new bone was formed on the periphery of the implant.

## Discussion

The ß-Mg_17_Al_12_ phase is the major precipitation phase in Mg-Al alloys, and it forms around grain boundaries. Because the grain boundaries do not remain constant at high temperatures, grain-boundary sliding occurs. Thus, the mechanical properties of these alloys degrade rapidly after heat treatments at temperatures higher than 120 °C^21^. The physical changes induced by such high-temperature treatments should therefore be evaluated with care. In a previous study, we found that the hydrothermal treatment of Mg at 160 °C for 4 h increased the corrosion resistance greatly. Most of the natural oxide film was peeled off after a hydrothermal treatment at temperatures higher than 200 °C for 3 h^16^. In this study, the group that was hydrothermally treated at 150 °C had the highest corrosion resistance. But, the samples of hydrothermally treated over 180 °C, the thick oxide film separated from the alloy. Thus, the hydrothermal treatments were performed at 120 and 150 °C.

PEO is a commonly used method for forming an oxide film on metallic materials by spark generation^22^ and results in formation of uniform and thick films. Through this process, it is possible to control the ion absorption rate of biodegradable Mg alloys, which makes them suitable for additional surface treatments. Magnesium and oxygen ionize and transform into Mg^2+^ and O^2−^ under high voltage. MgO is formed by outward migration of Mg^2+^ from substrate metal to discharge channels and inward migration of O^2−^ under high temperatures such as the reaction in (3).

In the first step, the formation mechanism of the oxide film by PEO treatment involves simultaneous formation at the film/electrolyte and substrate/film interface with the following steps:

1 $${\rm{Mg}}\to {{\rm{Mg}}}^{2+}+2{{\rm{e}}}^{-}$$

2 $${{\rm{Mg}}}^{2+}+2{{\rm{OH}}}^{-}\to {\rm{Mg}}{({\rm{OH}})}_{2}$$

3 $${{\rm{Mg}}}^{2+}+{{\rm{O}}}^{2-}\to {\rm{MgO}}$$

For anodization with Na_3_PO_4_, a dense, porous, and thick oxide film formed. Because the energy between the specimen being treated and the electrolyte was high due to the high-voltage plasma, irregular and fine cracks formed in the specimen. Mg_3_(PO_4_)_2_ was formed by Mg^2+^ from substrate material to discharge channels and inward migration of PO_4_^3−^from an electrolyte to discharge channels during PEO, such as the reaction shown in (4)^23^.

4 $$3{{\rm{Mg}}}^{2+}+2{{{\rm{PO}}}_{4}}^{3-}\to {{\rm{Mg}}}_{3}{({{\rm{PO}}}_{4})}_{2}$$

The electrodeposited Ca was less adherent and non-uniform on the surface, and the crystal phase of the dense absorption of Ca ions decreased the surface roughness more than the PEO layer. Furthermore, they had a sponge-like structure that contained nanopores. The films were formed with a low applied voltage (20 V). During electrodeposition, the excess applied current increased the potential, which may have caused inhomogeneities and defects in the deposited layer due to vigorous hydrogen evolution on the surface. Electrodeposition of calcium from Ca(NO_3_)_2_ resulted in the formation of a homogeneous Ca(OH)_2_ layer, as shown in the reaction described in (5)^24^.

5 $${\rm{Ca}}{({{\rm{NO}}}_{3})}_{2}+2{{\rm{H}}}_{2}{\rm{O}}\to {\rm{Ca}}{({\rm{OH}})}_{2}+2{{\rm{HNO}}}_{3}$$

After the hydrothermal treatment, Ca(OH)_2_ was dehydrated to CaO, which may have resulted from high temperature and pressure through the following steps:

6 $${\rm{Ca}}{({\rm{OH}})}_{2}\to {\rm{CaO}}+{{\rm{H}}}_{2}{\rm{O}}$$

Then, the process for HA-like substances can occur through the following steps:

7 $${{\rm{Ca}}}^{2+}+{{{\rm{HPO}}}_{4}}^{2-}+2{{\rm{H}}}_{2}{\rm{O}}\to {{\rm{CaHPO}}}_{4}\cdot 2{{\rm{H}}}_{2}{\rm{O}}\quad \quad ({\rm{brushite}})$$

8 $$10{{\rm{Ca}}}^{2+}+6{{{\rm{PO}}}_{4}}^{3-}+2{{\rm{OH}}}^{-}\to {{\rm{Ca}}}_{10}{({{\rm{PO}}}_{4})}_{6}{({\rm{OH}})}_{2}\quad \quad ({\rm{HA}})$$

9 $$6{{\rm{CaHPO}}}_{4}+4{\rm{Ca}}{({\rm{OH}})}_{2}\to {{\rm{Ca}}}_{10}{({{\rm{PO}}}_{4})}_{6}{({\rm{OH}})}_{2}+6{{\rm{H}}}_{2}{\rm{O}}$$

The group that had a hydrothermal treatment at 120 °C showed the disappearance of the Ca(OH)_2_ phase in addition to the formation of brushite with an acicular structure. The Ca(OH)_2_ phase could be transformed into HA or brushite (CaHPO_4_·2H_2_O) through deposition in SBF. The hydroxide coating contained only Ca^2+^ and OH^−^ ions. However, if it were to combine with Mg_3_(PO_4_)_2_, it would result in the formation of amorphous Ca-P clusters, which could act as seed crystals for HA and brushite^24^. Brushite, which is a type of calcium phosphate dehydrate, is monoclinic and has a higher solubility compared to other calcium phosphate phases. It is also more stable than HA^25^. The structure of the film formed by the hydrothermal treatment at 150 °C included crystallization, which resulted in the formation of hexagonal flakes and crystals with a petal-like structure. The hexagonal flakes included magnesium hydroxide (Mg(OH)_2_). As the temperature increased, the existing crystals dissolved slowly, recrystallized, and changed shape because of the resulting increase in pressure^16^. The petal-like crystals consisting of HA and OH^−^ generated in a hydrothermal treatment were based on Mg-OH and/or Ca-OH structures that formed nucleation sites for the HA^26^.

The deposited Ca penetrated the oxide film via the hydrothermal treatment. MgO/Mg(OH)_2_, due to the presence of magnesium, reacted with water by a hydrothermal system within the Ca-P layer. During a hydrothermal treatment at high temperature and pressure, the ionization constant of H_2_O increased, and water and Mg penetrated through the Ca-P layer. It resulted in the formation and growth of thick crystals of MgO/Mg(OH)_2_^16^.

From the graphs obtained by the potentiodynamic polarization tests, the Tafel slope could be determined and used to calculate the current density. By extrapolating these partial curves and determining their crossover point, the corrosion rate can be determined^27^. The thickness of the film from Ca deposition (Fig. 3(b)) did not increase compared to the film made through anodization (Fig. 3(a)), but the corrosion resistance increased since the porous surface of the anodic film was sealed by the formation of homogeneous Ca(OH)_2_ in the pores of anodic film.

The sample that was hydrothermally treated at 150 °C showed the lowest current density and a low corrosion rate. The anodic curve of the hydrothermally treated group increased rapidly and then decreased, which indicated there was formation and destruction of a passive film. In particular, the group hydrothermally treated at 150 °C formed a remarkably passive film, which exhibited low corrosion as well after the passive film was destroyed. The corrosion potential value at the parallel potential point was also the highest for the group that was hydrothermally treated at 150 °C. The passive film, which directs the formation energy of the natural oxide film, was lower than the energy of the corrosion of the deposited film. When the corrosion potential is applied over a stable passive potential area, the thickness of the passivation film is increasing^28^. Therefore, it was fitting that the group that was hydrothermally treated at 150 °C formed the most stable oxide film.

EIS is a nondestructive method that results in minimal changes in material properties due to a smaller width of frequency by AC signal that was applied to the material. The polarization resistance (R*p*) can be obtained from the Nyquist plot, which is the radius of the circular arc^29^. The circular arcs for the Ca deposition group and the group that was hydrothermally treated at 120 °C were distorted and noisy in the low-frequency area and were not ideal semicircles. The radius of the circular arc, which was related to the impedance, was not smooth. In the cases in which heterogeneities existed at the interface, a double layer was formed. The growth of an oxide film through the hydrothermal treatment method resulted in chemical bonding between the film and the substrate. Such films exhibited greater adhesiveness, which resulted in an increase in the corrosion resistance. In particular, since the thickness of the MgO layer formed on the group that was hydrothermally treated at 150 °C was 20 μm and because a dense Ca-P layer was formed, the corrosion resistance was higher compared to the group that was hydrothermally treated at 120 °C.

After immersion in Hank’s solution, spherical structures formed all over the film on the anodization group, which became more dense and increased in size. However, cracks also formed. The formed crystal phase consisted of Mg(OH)_2_. Ca deposition (b) resulted in sponge-like Ca(OH)_2_ crystals, which reacted with the P ions in Hank’s solution and became CaHPO_4_·2H_2_O crystals shaped like budding flowers. However, an increase in the Mg concentration and a decrease in the Ca concentration on the surface meant that the formation rate of MgO was higher than the formation rate for apatite. However, the corrosion resistance was not high. The samples in the hydrothermally treated groups exhibited Ca-P structures with different shapes. The Ca-P precipitates formed by the reaction with Mg(OH)_2_ included brushite (dicalcium phosphate dehydrate (CaHPO_4_·2H_2_O)), octacalcium phosphate (Ca_4_(HPO_4_)(PO_4_)_2_·25H_2_O), and HA^30^. The group that was hydrothermally treated at 120 °C exhibited a high-intensity CaHPO_4_·2H_2_O peak. Furthermore, brushite is more soluble than HA or the other calcium phosphate phase^25^ and was thus able to combine rapidly with body fluids. On the other hand, the group that was hydrothermally treated at 150 °C showed the coexistence of CaHPO_4_·2H_2_O and HA. The crystallized Mg(OH)_2_ did not show any structural changes and may have caused the bioactivity to become lower.

The MC3T3-E1 osteoblast cells from mouse calvaria have metabolic features similar to osteoblasts, including similar cell proliferation rates and differentiation and calcification characteristics during bone formation^31^. The anodization group without Ca deposition showed the lowest cell activity after 1 day of culture. The high concentration of P can alter the functioning of all body systems, including that of the musculoskeletal and cardiovascular systems^32^. The Ca deposition group showed cell activity similar to normal cells after 1 day. However, after 3 days, it showed low cell activity as well as low cell differentiation since the Ca deposition increased the pH of the surrounding environment and resulted in the formation of hydrogen gas due to the combination with OH^−^ ions^33^. The 150 °C group, which had the highest corrosion resistance, showed unexpectedly low cell activity because the HA crystals formed on its surface were very stable and they were not as soluble in the body as amorphous calcium phosphate^30^. Thus, the crystallization of the surface and its high energy reduced the cell activity and their degree of attachment.

Because of the various free radical groups of oxygen, H_2_O_2_ was found in large amounts in cells. It exhibited toxicity and could oxidize the various materials in the cells, such as proteins, DNA, and RNA and destroy its structure^34^. Thus, it was a good measure for evaluating the oxidation stress experienced by cells. After 1 day of cell culture, except for the hydrothermally treated at 120 °C group, all the groups exhibited high rates of H_2_O_2_ production, which indicated that the cells experienced high oxidation stress. On the other hand, after 3 days, the 150 °C group exhibited a decrease in the amount of H_2_O_2_ that was produced. Furthermore, the cell growth rate stabilized and was not significantly different from normal cells. The amount of H_2_O_2_ produced initially was related to the results of ALP activity, which was an enzyme associated with osteoblasts. The higher production of H_2_O_2_ disturbed the differentiation in osteoblasts, and the group that was hydrothermally treated at 150 °C exhibited an increase in cell differentiation after 3 days due to the decrease in the amount of H_2_O_2_ that was produced.

Remodeled bone consists of osteoblasts, while osteoclasts are involved in bone absorption. Osteoblasts control the activity and differentiation of osteoclasts^35^. The differentiation from osteoblasts to osteoclasts can occur after combination with RANKL and acceptor of RANKL expressed in osteoclast progenitors. Thus, the ions eluted from the surface-treated materials stimulated the osteoblasts, while the various local factors that were secreted probably affected the differentiation of the osteoclasts.

TRAP is a biomarker for osteoclast expression, which resulted in the generation of mononuclear precursor cells at the start of differentiation. However, when the cells are fused and differentiated into multinuclear matured osteoclast cells, they become attached to the bone surface and form a ruffled membrane. This membrane was dyed red in the sample anodized with Na_3_PO_4_ (Fig. 8A(a))^36^. On the other hand, after co-culture, the normal cells were differentiated into osteoblast cells. The RAW 264.7 cells had a structure similar to mononuclear precursor cells and failed to differentiate into osteoclast cells. All the groups caused the differentiation of multinuclear mature osteoclast cells, and the number of mononuclear osteoclast cells increased. Thus, the ions eluted from all the groups affected the differentiation of the osteoclast cells. However, the Ca-P layer in the case of the heat-treated groups inhibited the differentiation of osteoclast cells.

In the initial period after the placement of the implants in the rat tibiae (approximately 3 weeks), the elution rate of Mg ions decreased with coating thickness. A gas formed in the bone marrow, which is where fluid flow occurs. This gas was absorbed into the body after 6 weeks. Therefore, the corrosion rate could be controlled only in the initial period after implant placement. H_2_, which was the main component of the gas, does not affect apoptosis. However, it interferes with initial bone formation and impairs the bonding between the implant and bone. The initial corrosion rate was reduced by the hydrothermal treatment successfully because the jacket of bubbles around the bone marrow was not observed after 3 weeks. The cell stress level and osteoclast formation rate as determined *in vitro* were the lowest in the group that was hydrothermally treated at 120 °C, which contained highly soluble brushite. On the other hand, the group that was hydrothermally treated at 150 °C had the thickest coating and showed the highest corrosion resistance. Finally, a high amount of new bone formation was observed in the rat tibia.

The Ca-P layer in the group that was hydrothermally treated at 120 °C promoted new bone formation due to the initial delay in corrosion and the higher bone cell activity during implantation for 3 weeks. On the other hand, after 3 weeks, the Ca-P layer resulted in localized corrosion because of the destruction of the layer, which resulted in the elution of Mg ions. However, even though the changes in the Ca-P layer with the hydrothermal treatment temperature resulted in differences in bone cell differentiation, new bone formation occurred in the bone marrow, which confirmed that a stable and refined film was formed by the hydrothermal treatment.

## Materials and Methods

### Material preparation

The Mg-Al-Zn-Ca alloy (KMTRA, Korea) used was cast from a commercial AZ31B alloy and 1.5 wt% granule-type Ca. The cast alloy was extruded at 650 °C to obtain a sheet with a width of 160 mm and thickness of 50 mm (this was achieved by rolling a sheet with width of 80 mm and thickness of 50 mm twice), as per procedures reported previously^37^. The sheet to be subjected to anodic oxidation was cut in pieces having dimensions of 15 × 15 × 2 mm. Further, 1.5 × 1.5 × 4 mm samples of the Mg-3Al-1Zn-1.5Ca alloy were also prepared for histological analysis using rats.

### Surface treatment

For the PEO treatment, a platinum plate and the sample to be treated were connected to the cathode and anode, respectively, of a direct current power supply (Kwangduk FA, Korea). A preliminary test was conducted in an electrolytic solution consisting of 1.0 M sodium hydroxide (NaOH), 0.10 M glycerol, and 0.10 M sodium phosphate (Na_3_PO_4_) at 300 mA/cm^2^. The pulse width was set to 300 ms, and the duty cycle was fixed at 50% for 3 min.

For the electrochemical deposition process, the alloy sample and the platinum plate were used as the cathode and anode, respectively, in contrast to the anodization process. The distance between the anode and cathode was 20 mm and the electrolyte was 0.164 M Ca(NO_3_)_2_·4H_2_O. The experiment was conducted at a constant voltage as 20 V for 2 min at ambient temperature. Next, the sample was washed lightly in distilled water and then dried at 37 °C.

After the PEO coating and electrochemical deposition processes, the sample was placed in an autoclave (Ilshin Autoclave Co, Ltd, Korea) and heated at 3 °C/min to 120 and 150 °C in distilled water for 2 h at a vapor pressure 8.8 MPa to induce the precipitation of Ca-P crystals on its surface.

### Characterization and evaluation of corrosion resistance

The microstructural morphologies and cross-sections of the films formed after the various treatments were observed by SEM (JSM-6400, JEOL, Japan). The concentrations of the elements present on the surface and in the cross-section of the film were determined by EDS (7274, Oxford Instruments, England). The phase of the surface layer was analyzed by X-ray diffraction (XRD) analysis (Dmax III-A type, Rigaku, Japan) using a Cu target. The step size was 0.0334°, and the time per step was 50 s.

The electrochemical impedance spectroscopy (EIS) and potentiodynamic polarization measurements were performed to evaluate the corrosion performance of the coating using a potentiostat/galvanostat (2273, AMETEK, USA). A Ag/AgCl/KCl electrode (saturated) was used as the reference electrode, and a platinum electrode was used as the counter electrode; the specimen was connected to the working electrode. The corrosion potential and corrosion current density were measured by a scanning rate of 3 mV/s in a 0.9% NaCl solution. The EIS measurements were made at frequencies of 100 mHz to 100 KHz.

### Immersion test in similar body fluid

The samples were immersed in a similar body fluid (SBF) for 7 days, in order to evaluate their biological activities. The SBF used was Hank’s balanced salt solution (H2387, Sigma Chemical Co, USA), which consisted of 0.185 g/l calcium chloride dihydrate, 0.09767 g/l magnesium sulfate, and 0.350 g/l sodium hydrogen carbonate. The pH of the Hank’s solution was adjusted to 7.4 using NaOH and HCl solutions before the experiments. The Hank’s solution was changed every 48 h, in order to keep the concentrations of the ions constant, as is the case with body fluids. After the test, the samples were cleaned in distilled water ultrasonically and were observed using SEM (JSM-5900, JEOL, Japan) and EDS (Oxford, England) to determine the elemental concentrations and by XRD analysis (Dmax III-A type, Rigaku, Japan) to determine the phases present.

### Cytotoxicity assessment

#### Osteoblast cell preparation

Osteoblast cells (MC3T3-E1) were purchased from the American Type Culture Collection. Next, 10% fetal bovine (FBS, Gibco Co., USA), 500 unit/ml of penicillin (Gibco Co., USA), and 500 unit/ml of streptomycin (Gibco Co., USA) were added to *α*-MEM (Gibco Co., USA) to prepare the culture medium. Incubation was carried out at 37 °C in an atmosphere containing 5 vol% CO_2_ for 20 h. Cell extraction was done in an extraction medium with *α*-MEM/sample volume ratio of 4:1 in a humidified atmosphere with 5% CO_2_ at 37 °C for 48 h. The *α*-MEM medium was used for the normal group, whereas *α*-MEM containing 100 μM H_2_O_2_ was used as the positive control group.

#### Cell proliferation and differentiation

The cells were incubated in cell culture plates at 2.5 × 10^4^ cells/well in 48 wells and incubated for 20 h to allow for cell attachment. The medium was exchanged with the extraction medium of the material. After incubation in a humidified atmosphere for 1 and 3 days, the cultured cells were stained with 0.3% crystal violet to allow for observations of the cell morphology using optical microscopy (DM2500, Leica, Japan). Cellular cytotoxicity was determined using a standard water-soluble tetrazolium salt (premix WST-1 kit) (MK400, Takara, Japan) as a cell proliferation assay for colorimetric analyses. The cell proliferation reagent, WST-1, was added in an amount of 100 μl/well to 96 wells, and the cells were incubated further at 37 °C for 1 h. The formazan dye intensity was subsequently measured using a microplate spectrophotometer (EMax, Molecular Devices, USA) at a wavelength of 450 nm.

#### Hydrogen peroxide (H_2_O_2_) assay

After incubation in a humidified atmosphere for 1 and 3 days, the amounts of H_2_O_2_ produced from the culture medium extracted from the samples at the indicated times were measured using a hydrogen peroxide assay kit (Biovision Research Products, USA). The absorbance was measured at 570 nm using a microplate spectrophotometer (EMax, Molecular Devices, USA). To determine the H_2_O_2_ concentrations, 0–10 nmol H_2_O_2_ standards were prepared, and the standard curve was plotted. The H_2_O_2_ contents in the samples were determined through a comparison with the predetermined H_2_O_2_ standard curve.

#### Alkaline phosphatase (ALP) activity

For colorimetric analyses, cells were washed with 0.9 wt% NaCl after cultured for 8 days. Then, an extraction solution (saline solution containing 1% NP-40) was added to each well for the solubilization of the adherent cells. Next, a substrate solution (0.2 M Tris-HCl buffer with p-nitro-phenyl phosphate (pNPP)) was added and allowed to react at 37 °C for 60 min using an ALP assay kit (MK301, Takara, Japan). The absorbance was measured at 405 nm using a microplate spectrophotometer (EMax, Molecular Devices, USA).

#### Osteoclast formation and activity

MC3T3-E1 cells and RAW 264.7 murine monocytic macrophage cells (5:1 ratio) were co-cultured at 2.5 × 10^4^ cells/well in 48 wells in *α*-MEM (Gibco Co., USA) with 10% FBS (Gibco Co., USA), 500 unit/ml of penicillin (Gibco Co., USA), and 500 unit/ml of streptomycin (Gibco Co., USA). Incubation was carried out at 37 °C in an atmosphere containing 5 vol% CO_2_ for 20 h. The extraction was carried out in an extraction medium with an *α*-MEM/sample volume ratio of 4:1 in a humidified atmosphere with 5% CO_2_ at 37 °C for 48 h. The *α*-MEM medium was used for the normal group, while *α*-MEM containing 100 μM H_2_O_2_ was used for the positive control group. In each well, the medium was exchanged with the extraction medium.

After incubation in a humidified atmosphere for 8 days, the co-cultured cells were washed with 0.9 wt% NaCl. Next, the extraction solution (0.9 wt% NaCl containing 1% NP-40) was added to the adherent cells for solubilization and subsequent colorimetric analysis. To assess the formation and activity of osteoclasts, the substrate solution (0.5 M sodium acetate with pNPP) and 0.5 M sodium tartrate were added to each well and made to react at 37 °C for 60 min using an TRACP assay kit (MK301, Takara, Japan). The stop solution (0.5 N NaOH) was added for color formation, and the absorbance was measured at 405 nm using a microplate spectrophotometer (EMax, Molecular Devices, USA). The osteoclast cells were stained with a tartrate-resistant acid phosphatase (TRAP) staining kit (B-Bridge International, Inc., USA) and were observed by optical microscopy (DM2500, Leica, Japan).

#### Statistical analysis

The data were analyzed for statistical significance using the one-way ANOVA-test except for the negative group; a p value less than 0.05 was considered significant.

#### *In-vivo* analysis

A total of 16 male Sprague Dawley rats (270–280 g, Orient Bio Co., Korea) were used in this experiment, which was identifying the institutional approving the experiments by the local animal ethics committee of Chonbuk National University, South Korea (IRB: CBNU 2015-034), and the experiment was performed in accordance with relevant guidelines and regulations. During the experiments, 0.5 ml/100 g of ketamine (Ketamine HCl57.68 mg, Yuhan, Korea) was administered intramuscularly to the rats for anesthesia. A 2-mm-diameter drill was used to make a hole with a depth of 3 mm in the rat tibia. The samples (1.5 × 1.5 × 4 mm) were implanted in these holes. Two rats from each group were sacrificed 3 and 6 weeks after implant placement. The tibia sections with the implants cut and fixed in 10% formalin solution. They were then stained with the Villanueva Osteochrome bone staining solution (Polysciences Inc., USA) and embedded in poly(methyl methacrylate). The embedded blocks were sectioned and ground along the longitudinal axis of the implants to a thickness of approximately 10–40 μm. Finally, histological analyses were performed to examine for new bone between the tibia and the implant using optical microscopy (DM 2500 M, Leica, Germany).
